# Highly Efficient Candlelight Organic Light-Emitting Diode with a Very Low Color Temperature

**DOI:** 10.3390/molecules26247558

**Published:** 2021-12-13

**Authors:** Shahnawaz, Iram Siddiqui, Mangey Ram Nagar, Abhijeet Choudhury, Jin-Tin Lin, Dovydas Blazevicius, Gintare Krucaite, Saulius Grigalevicius, Jwo-Huei Jou

**Affiliations:** 1 Department of Materials Science and Engineering, National Tsing Hua University, Hsinchu 30044, Taiwan; shaan88usmani@gapp.nthu.edu.tw (S.); iramsidd29@gmail.com (I.S.); mangeyrnagar@gmail.com (M.R.N.); abhijeetchoudhury101@gmail.com (A.C.); s10030267@gmail.com (J.-T.L.); 2 Department of Polymer Chemistry and Technology, Kaunas University of Technology, Radvilenu Plentas 19, LT50254 Kaunas, Lithuania; dovydas.blazevicius@ktu.lt (D.B.); gintare.krucaite@ktu.lt (G.K.)

**Keywords:** phenoxazine, amorphous layer, efficiency, host derivative, light emitting diode

## Abstract

Low color temperature candlelight organic light-emitting diodes (LEDs) are human and environmentally friendly because of the absence of blue emission that might suppress at night the secretion of melatonin and damage retina upon long exposure. Herein, we demonstrated a lighting device incorporating a phenoxazine-based host material, 3,3-bis(phenoxazin-10-ylmethyl)oxetane (BPMO), with the use of orange-red and yellow phosphorescent dyes to mimic candlelight. The resultant BPMO-based simple structured candlelight organic LED device permitted a maximum exposure limit of 57,700 s, much longer than did a candle (2750 s) or an incandescent bulb (1100 s) at 100 lx. The resulting device showed a color temperature of 1690 K, which is significantly much lower than that of oil lamps (1800 K), candles (1900 K), or incandescent bulbs (2500 K). The device showed a melatonin suppression sensitivity of 1.33%, upon exposure for 1.5 h at night, which is 66% and 88% less than the candle and incandescent bulb, respectively. Its maximum power efficacy is 23.1 lm/W, current efficacy 22.4 cd/A, and external quantum efficiency 10.2%, all much higher than the CBP-based devices. These results encourage a scalable synthesis of novel host materials to design and manufacture high-efficiency candlelight organic LEDs.

## 1. Introduction

The white lighting sources with high color temperature consist of blue light enriched emission, responsible for blue hazards especially at dawn-, dusk-, and night-time, that may lead to serious human health disorders such as retinal cell damage and melatonin suppression, increasing insomnia, obesity, or even cancer risk [1,2,3,4,5,6,7,8,9,10]. Moreover, blue-emission can cause ecological disruptions as well as discoloring well-known paintings [11,12]. The same views about the dangers posed by the blue hazard have also been echoed by various governmental and scientific organizations [13,14,15,16].

The scientific community has been demanding more research in development of blue-emission less low color temperature lighting. Interestingly, the emission spectra of the candles and old incandescent light lamps have generally emitted moderately lower blue-emission. However, the flickering nature of the candles, along with the energy-inefficient nature of both light sources, resists the devices to be reintroduced in the commercial market [4,11,12,17]. To eradicate such issues, candlelight-style lighting sources were introduced.

The next-generation organic LED lighting systems can generate the blue-free emission candlelight-style lighting that provides a pleasant sensation for the users due to its glare-free Lambertian quality [1]. Candlelight-style organic LEDs are human and environmentally friendly due to the absence of blue emission that might suppress the secretion of melatonin and damage retina upon long exposure at night.

Jou and co-workers were one of the first to report a psychologically friendly organic LED lighting device with a power efficacy (PE) of 11.9 lm/W at 100 cd/m^2^ and external quantum efficiency (EQE) of 6.4% [18]. In 2017, they reported a low-cost solution-processable organic LED with a power efficacy of 7.2 lm/W at a low-color temperature of 1807 K [2]. There have been extensive research efforts to develop next generation-al human-friendly low color temperature devices.

To achieve highly efficient and cost-effective organic LEDs, different research groups have reported numerous effective host-guest device structures to overcome exciton quenching in the emissive region, enabling high-performance electroluminescent (EL) devices [19,20,21,22,23,24,25]. The host materials play an essential role in the overall EL characteristic of organic LEDs [26,27,28,29]. Developing a suitable host material is highly essential to pose following properties as (1) appropriate frontier energy-levels HOMO/LUMO for aligning host-guest molecules, helping in the formation and harvesting the radiative excitons in the emissive region, (2) efficient triplet energy-level corresponding to the guest molecules, assuring complete energy transfer from host to guest materials, (3) efficient charge transfer capabilities, improving the charge transfer and charge recombination in the host-guest system, and (4) high glass-transition temperature and thermal-decomposition temperatures to attain good thin-films morphology to realize highly stable organic LEDs. The carbazolyl, indolyl, and phenothiazinyl chromophores display enormous triplet energies and appropriate host materials for the dry-processed organic LED devices [20,30,31,32,33]. However, they encounter problems in solution-processed organic LEDs, inspiring us to search for novel solution-processable host materials with desired characteristics. 

Herein, we report easily synthesized and cheap phenoxazine-based host material synthesized by modest one-step reaction technique and utilized in the fabrication of solution-processed organic LED devices [34,35,36,37]. We fabricated solution-processable candlelight organic LED devices using host materials 3,3-bis(phenoxazin-10-ylmethyl)oxetane (BPMO) and 4,4′-Bis(N-carbazolyl)-1,1′-biphenyl (CBP) along with an orange-red tris(2-phenylquinoline)iridium(III) (Ir(2-phq)_3_) and a yellow emitter iridium(III) bis(4-phenylthieno[3,2–c]pyridinato-N,C2’)acetylacetonate (PO-01) resulting in a simpler device architecture. The BPMO-based device showed a maximum luminance (L_max_) of 14,950 cd/m^2^ (equivalent to 16,500 candles in an area of 1 m^2^) PE_max_ of 24 lm/W, current efficacy (CE_max_) of 22.4 cd/A and EQE_max_ of 10.2% with a low CT of 1690 K at a voltage (2.9 V). While CBP-based device displayed L_max_ 8393 cd/m^2^ with PE_max_ of 9.6 lm/W, CE_max_ 11.7 cd/A, EQE_max_ 6.8%, and CT as low as 1768 K at 3.5 V that is much lower than its counterpart.

Furthermore, the resulting solution-processed organic LED device is the first reported low CT with a record-break maximum permissible exposure limit (MPE) at 100 lx, 57,696 s comparative to a candle (2750 s) and an incandescent bulb (1100 s). Moreover, it exhibits 1.33% melatonin suppression sensitivity (MSS) upon exposure for 1.5 h at night at 100 lx, 66%, and 88% less than a candle and incandescent bulb, respectively.

## 2. Result and Discussion

### 2.1. Synthesis

**Scheme 1:**
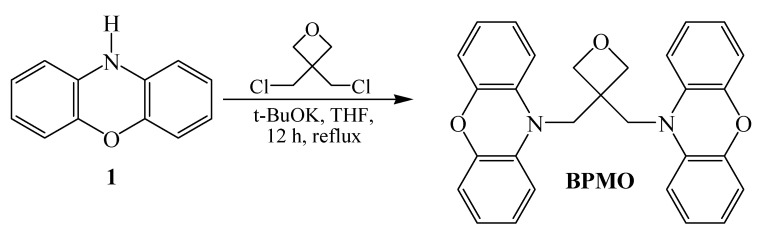
Synthesis route of phenoxazine-based compound BPMO.

The phenoxazine-based host material was prepared using an approach similar to our previously reported work [37] which was carried out by the simple one-step synthetic route as shown in Scheme 1. 3,3-Bis(phenoxazin-10-ylmethyl)oxetane (BPMO) host was obtained by N-alkylation reaction of phenoxazine (1) with 3,3-bis(chloromethyl)oxetane using potassium tert-butoxide in tetrahydrofuran (THF). Mass and NMR spectroscopy had recognized the presence of the newly synthesized derivative. The data were found to be well in line with the proposed structure (See details Section 3.1).

### 2.2. Theoretical Analysis

**Figure 1:**
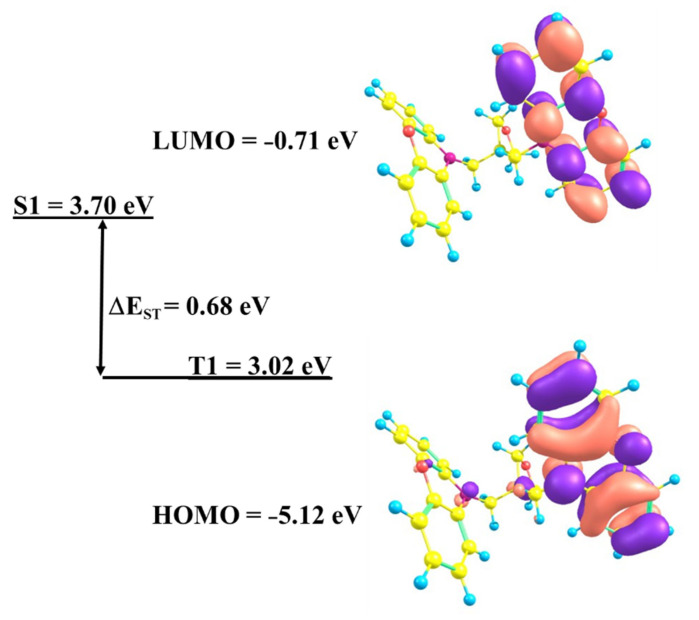
The HOMO (−5.1 eV), LUMO (−0.7 eV), and triplet energy (3.0 eV) of BPMO host theoretically calculated using DFT.

In order to better understand the link between photophysical and electronic characteristics of the synthesized host material BPMO, the theoretical calculation was carried out based on Gaussian software, density functional theory (DFT). Figure 1 shows the electron density distributions of the frontier molecular orbitals. The molecular structure is distributed by the highest occupied molecular orbitals (HOMO) and the lowest unoccupied molecular orbitals (LUMO). For BPMO, the HOMO/LUMO values estimated are −5.1/−0.7 eV, while the singlet and triplet energy are 3.7 and 3.0 eV, respectively (Table 1). Therefore, the BPMO host has 0.68 eV singlet-triplet splitting energy.

### 2.3. Thermal Characteristics

The as-reported material BPMO possesses a very high thermal stability and crystallinity. Its melting point as 198 °C and the glass transition temperature (T_g_) as 66 °C was recorded using (TGA). While (DSC) characterization confirmed the high crystallinity of the host materials [37] (See details Section 3.2).

### 2.4. Photophysical and Electrochemical Characteristics

**Figure 2:**
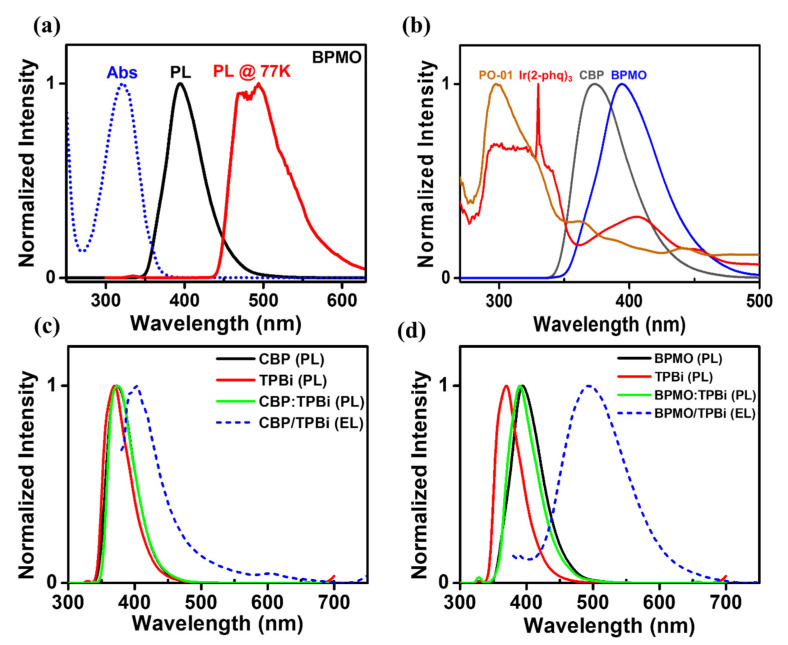
The photophysical and electroluminescent (EL) characteristics showing (**a**) singlet (3.44 eV) and triplet energy (2.87 eV) of the host BPMO measured using Abs/PL and PL/PL (at 77 K) spectrum, respectively. (**b**) The overlapping area between PL of hosts BPMO and CBP and Abs of yellow (PO-01) and orange-red (Ir(2-phq)_3_) dye. BPMO shows the larger overlapping area, (**c**) and (**d**) show a larger redshift between the PL and EL spectra for host BPMO than CBP. The shifting is observed between the individual and mixture of host BPMO/CBP and electron-transporting layer (ETL) TPBi, respectively.

Figure 2 shows the photophysical and electroluminescent (EL) characteristics of the host CBP and BPMO using UV-vis absorbance (Abs), photoluminescence (PL), and low-temperature phosphorescence (LTPL) characterizations. Abs, PL, and LTPL (at 77 K) spectra were observed at 320, 395, and 495 nm, respectively. The optical energy bandgap (E_g_) of 3.85 eV for BPMO was estimated by absorbance peak. Figure 2a shows the singlet (3.44 eV), and triplet energy (2.87 eV) (see Table 1) of the host BPMO calculated using the intercepting wavelength of Abs: PL (360 nm) and Abs: LTPL (436 nm). The formula for calculating singlet and triplet energy is

Singlet: 1240/intercepting wavelength of UV-vis: PL

Triplet: 1240/intercepting wavelength of UV-vis: LTPL

Figure 2b shows the overlapping area between normalized PL of hosts BPMO and CBP and normalized Abs of yellow (PO-01) and orange-red (Ir(2-phq)_3_) dye incorporated in the candlelight organic LED devices. BPMO shows the larger overlapping area with the absorbance of yellow dye (PO-01) and orange-red dye (Ir(2-phq)_3_) as 20.37 and 31.52 square units, respectively, in lieu of host CBP (19.36 and 22.6 square units).

Furthermore, Figure 2c,d shows a more significant redshift between the PL and EL spectra for host BPMO than CBP. The shifting is observed between the individual (BPMO/CBP and TPBi) and mixture (BPMO/CBP:TPBi) of the host and electron-transporting layer (ETL). A comparative redshift suggests the possibility of exciplex formation between the host BPMO and ETL TPBi (See details Section 3.2)

Figure S1 shows the cyclic voltammetry (CV) curve of BPMO in dichloromethane (DCM) for the oxidation scan. The HOMO and LUMO were calculated as −5.39 eV and −1.54 eV, respectively, from the CV curve, using the optical energy bandgap (E_g_) 3.85 eV.

Table 1 represents the photophysical and electrochemical characteristics of the host BPMO and commercial host CBP [38,39,40] (See details Section 3.2).

### 2.5. Charge Transporting Properties (HOD/EOD)

The charge transport characteristics of the host and guest materials play a vital role in deciding effective organic LEDs performance. The hole-only and electron-only devices were fabricated based on CBP and BPMO hosts to determine their carrier mobilities. 

**Figure 3:**
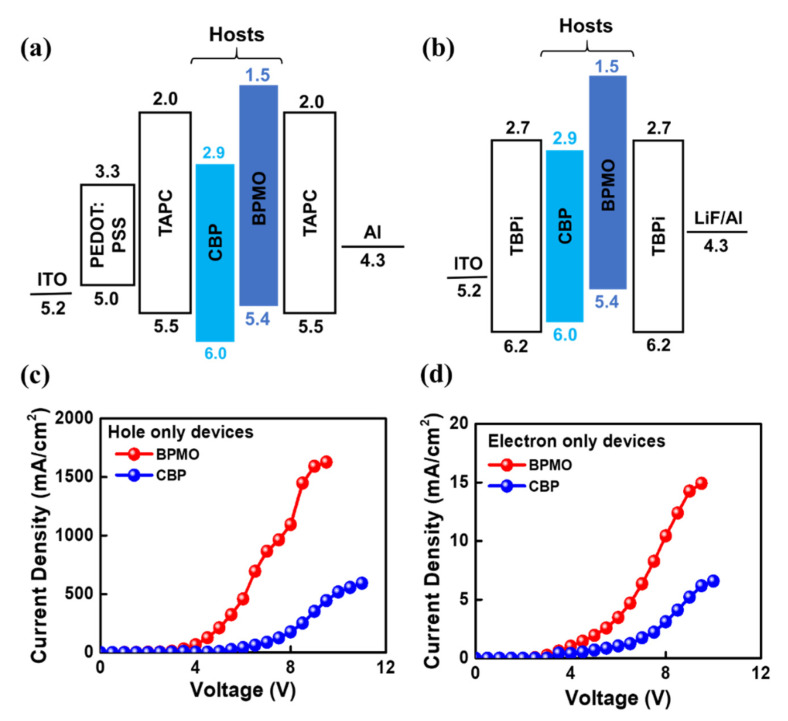
Schematic energy level diagrams of (**a**) hole-only, (**b**) electron-only devices, current-density-voltage curves of BPMO- and CBP-based (**c**) hole-only devices, and (**d**) electron-only devices. The cures reveal that BPMO host composing device has better charge-carrier mobility than CBP.

Figure 3a shows the schematic energy level diagrams of hole-only and electron-only devices. The devices were configured as

Hole-only device: ITO (125 nm)/PEDOT:PSS (35 nm)/TAPC (20 nm)/CBP or BPMO (20 nm)/TAPC (20 nm)/Al (200 nm)

Electron-only device: ITO (125 nm)/TPBi (35 nm)/CBP or BPMO (20 nm)/TPBi (40 nm)/LiF (1 nm)/Al (200 nm).

Figure 3c,d shows the current-density-voltage curves of BPMO- and CBP-based hole-only devices and BPMO- and CBP-based electron-only devices, respectively. The current density-voltage results from the hole-only device suggest that BPMO displays much better (almost 3 times) hole-transporting characteristics than CBP. Moreover, from an electron-only device, BPMO displays better electron-transporting properties as compared with the CBP. 

Moreover, BPMO shows bipolar nature, i.e., the hole and electron current densities are comparably equivalent, indicating balanced charge transport in organic LEDs.

### 2.6. Electroluminescent Properties 

**Figure 4:**
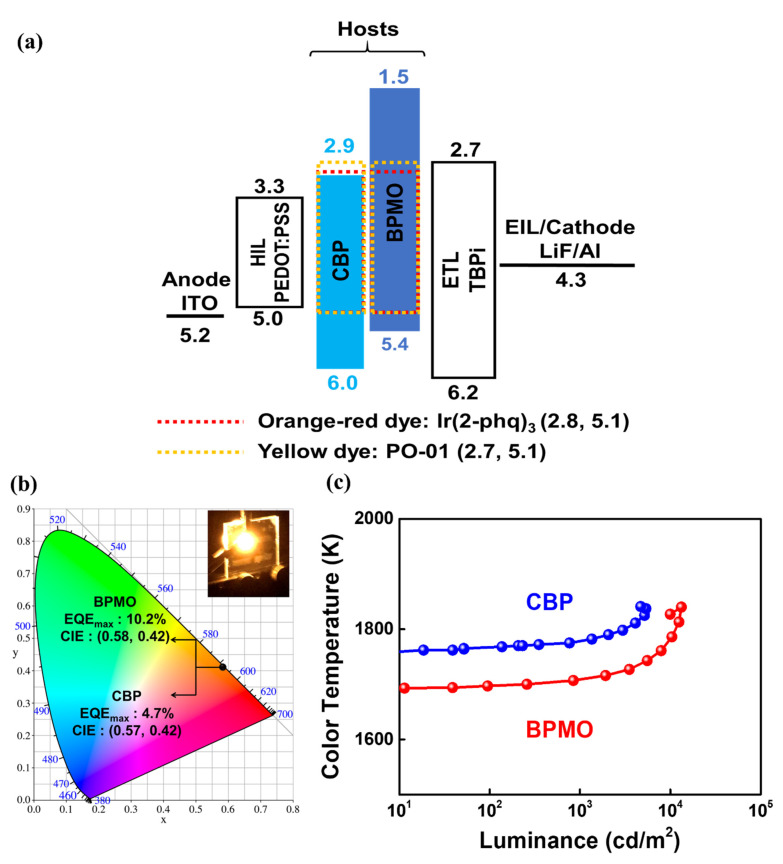
BPMO- and CBP-based candlelight organic LED showing (**a**) schematic energy level diagram using emitters PO-01 (yellow) and Ir(2-phq)_3_ (orange-red), (**b**) CIE chromaticity with achieved maximum EQE and device pixel image (inset figure), and (**c**) color-temperature variation with luminance.

Solution-processed candlelight organic LED devices using host materials BPMO and CBP had been fabricated. Figure 4a shows the schematic energy level diagram using emitters PO-01 (yellow) and Ir(2-phq)_3_ (orange-red) for BPMO- and CBP-based candlelight organic LED. The device structure is configured as ITO/PEDOT:PSS/BPMO or CBP: PO-01 (10 wt%): Ir(2-phq)_3_ (x wt%) (x = 7.5, 10.0, 12.5, 15.0)/TPBi/LiF/Al. The materials utilized and the device fabrication are discussed in Schemes S3 and S4, respectively.

Figure 4b shows the CIE chromaticity coordinates for BPMO-based device as (0.58, 0.42) and CBP-based device as (0.57, 0.42). The corresponding maximum EQE (EQE_max_) achieved were 10.2% and 4.7%, respectively. The device pixel image (inset figure) shows the candlelight emission.

Figure 4c shows the color-temperature (CT) variation with luminance for BPMO- and CBP-based devices. It can be seen that for the same luminance, CBP-based shows higher CT than BPMO-based device. The color temperature varies from 1690 to 1827 K for BPMO-based, while from 1751 to 1841 K for CBP-based candlelight organic LED, indicating the usefulness of BPMO as a host for candlelight organic LED.

The electroluminescent properties were studied for BPMO- (Figure S3a) and CBP-based (Figure S4a) candlelight organic LED devices. The yellow emitter (PO-01) at 10 wt% and orange-red emitter (Ir(2-phq)_3_) were utilized and doped with different concentrations, as shown in Table S1. 

It is observed that the EL spectra showed a bathochromic shift on increasing the concentration of orange-red emitter from 7.5 to 15 wt% (Figures S3b and S4b). For BPMO-based devices, the maximum power efficacy (PE_max_) changes from 22.1 to 19.9 lm/W, current efficacy (CE_max_) from 20.3 to 18.2 cd/A, external quantum efficiencies (EQE_max_) from 9.2 to 8.5%, and CT from 1730 to 1705 K. While CBP-based devices exhibit changes in PE_max_ from 9.3 to 7.6 lm/W, CE_max_ from 11.8 to 9.3 cd/A, EQE_max_ from 5.0 to 4.0%, and CT from 1790 to 1723 K (Figures S3c–d and S4c–d). It is observed that the efficiencies and CT are concentration dependent. Therefore, the optimized concentration is 10 wt% for the orange-red emitter. Furthermore, we can reduce the candlelight color temperature by incorporating a higher concentration of orange-red emitter, utilizing highly efficient orange-red emitter, and balanced charge recombination in the emission zone [41,42,43].

Table 2 shows the power efficacy (PE), current efficacy (CE), EQE, color-temperature (CT) of studied BPMO- and CBP-based candlelight organic LED using (at 10 wt%) yellow (PO-01) and orange-red (Ir(2-phq)_3_) emitters at 100, 1000 cd/m^2^, and the peak efficiency luminance (at max). The BPMO-based device shows a high luminance of 14,950 cd/m^2^ with PE_max_ of 24 lm/W, CE_max_ of 22.4 cd/A, and EQE_max_ of 10.2% at a very low voltage (2.9 V). At 100 cd/m^2^, a 22.0 lm/W PE, 22.4 cd/A CE, 10.2 % EQE, 1690 K CT is obtained at 3.2 V. Even at higher luminance, i.e., at 1000 and 10,000 cd/m^2^, the efficiencies are pronounced indicative of low roll-off at a very low CT. The results may be attributed to the balanced-charge transport, aligned HOMO, LUMO, and triplet energies, a low hole-injection barrier between HIL and emissive layer (EML), and a large hole-injection barrier between EML and ETL.

**Figure 5:**
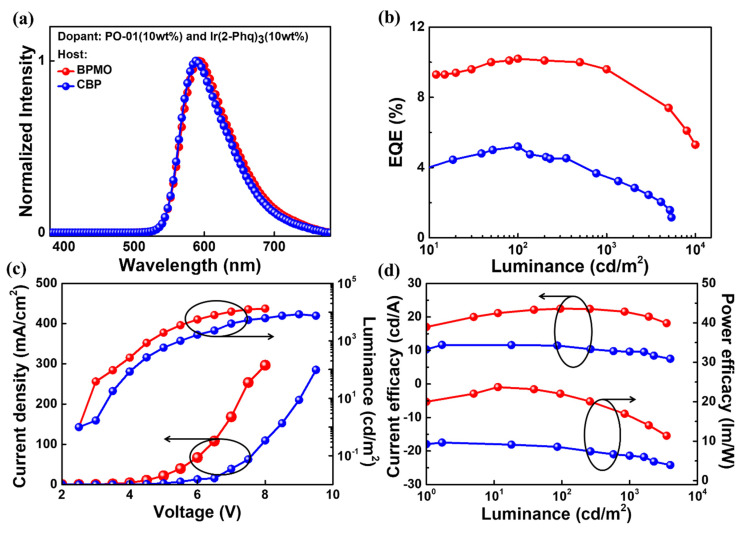
The studied BPMO- and CBP-based candlelight organic LEDs with (at 10 wt%) yellow and (at 10 wt%) orange-red emitter showing (**a**) the electroluminescent spectra, (**b**) variation of EQE with respect to luminance, (**c**) luminance-voltage-current density (L–V–J), (**d**) power efficacy-luminance-current-efficacy curve. The BPMO-based organic LED device shows better performance compared to the CBP-based device.

While CBP-based device displayed luminance as high as 8393 cd/m^2^ with PE_max_ of 9.6 lm/W, CE_max_ 11.7 cd/A, EQE_max_ 6.8%, and CT as low as 1768 K at 3.5 V that is much lower than its counterpart in all respect suggesting BPMO-based devices are 150, 91, and 50% better in terms of PE, CE, and EQE, respectively (Figure 5b,d).

Figure 5c shows the current-density curves concerning voltages. The BPMO-based device possesses higher current density and luminance than the CBP-based device, indicative of the high carrier mobility of BPMO. Such devices may show a better injection of positive charge carriers and efficient exciton generation in the emissive zone [44,45]. However, increasing the voltage increases the current density and luminance decreases due to charge imbalance and the influence of exciton quenching [46]. 

Figure 5b displays the external quantum efficiencies curves concerning luminance for the candlelight organic LED devices having different hosts, i.e., BPMO and CBP. Candlelight organic LED devices fabricated with BPMO possesses higher EQE as compared with control device fabricated using CBP. Moreover, it can be observed that the EQE increases as the luminance increases up to a certain level, and afterward, EQE starts decreasing because of charge imbalance and exciton quenching [47,48,49]. Figure 5a exhibits electroluminescence spectra of candlelight organic LED fabricated with different host BPMO and CBP. It is observed that the EL spectra of BPMO-based candlelight organic LED are slightly red-shifted as compared with CBP-based devices, which is attributed to high hole mobility of BPMO and formation of excitons at ETL/EML interface [50,51,52].

**Figure 6:**
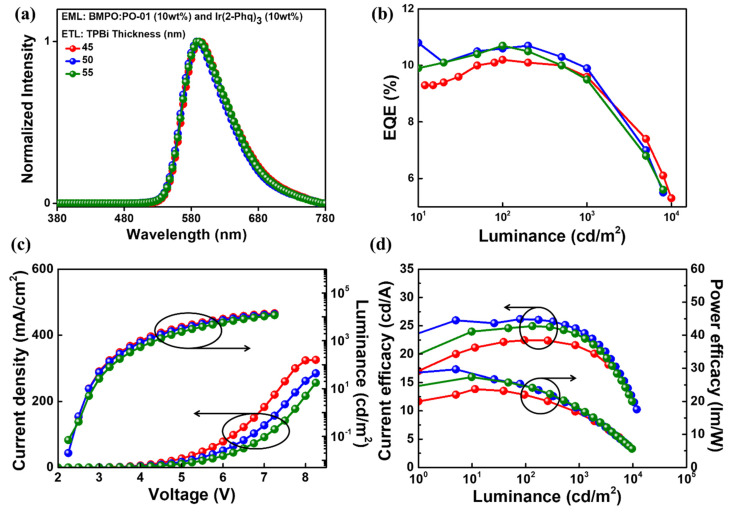
The studied BPMO- and CBP-based candlelight organic LEDs with (at 10 wt%) yellow and (at 10 wt%) orange-red emitter by varying electron transporting layer (ETL) thickness showing (**a**) the electroluminescent spectra, (**b**) variation of EQE with respect to luminance, (**c**) luminance-voltage-current density (L–V–J), (**d**) power efficacy-luminance-current-efficacy curve. The device with 55 nm thickness shows better performance among all.

For achieving higher efficiency, the device is optimized by varying the thickness of the electron-transport layer (ETL). The EL properties of studied organic LED devices are shown in Figure 6, and the values are summarized in Table 3. Figure 6 shows the studied BPMO- and CBP-based candlelight organic LEDs with (at 10 wt%) yellow and (at 10 wt%) orange-red emitter by varying electron transporting layer (ETL) thickness. Negligible change is observed in EL spectra on varying thickness from 45–55 nm (Figure 6a). Figure 6b displays that EQE increases on increasing the thickness, which may be attributed to microcavity changes in the fabricated organic LED device.

Figure 6c shows that the thicker the device is, the lower is the current density and luminance attributed to the imbalanced charge carriers that may cause a bulk majority carrier leading to non-radiation recombination [53,54,55]. 

Figure 6d demonstrates that the PE and CE meaningfully changed depending on the thickness of electron transport layers [56,57,58], i.e., increases with increasing the thickness of ETL. The PE_max_ varies from 23.1 to 24.8 lm/W and CE_max_ from 22.1 to 28.8 cd/A as the thickness of the ETL increases from 40 to 50 nm, which may be due to balanced charge-carriers in the emissive region. However, further increasing the thickness to 55 nm, a drop in PE and CE may be attributed to variations in trap densities in the ETL that may limit the charge transport and cause the charge imbalance in the emissive region [59,60,61].

Moreover, the color temperature of candlelight organic LED increases from 1690 to 1785 K as the thickness increases from 45 to 50 nm, which may be attributed to the changes in the recombination zone position in the emissive layer [62,63]. 

### 2.7. Comparison between the Studied Very Low Color-Temperature Candlelight Organic LED and Commercial Luminaires 

Table 4 shows the comparison between the spectrum, color temperature (CT), melatonin suppression sensitivity (MSS) (1.5 h exposure), and maximum permissible exposure limit (MPE) of the studied very low color temperature candlelight organic LED and the commercial luminaires, including, incandescent bulbs, warm white LEDs, and organic LEDs, cold white LEDs, and organic LEDs, and CFLs. 

The blue-emission-free BPMO-based candlelight organic LED possesses a color temperature of 1690 K, 180 times friendlier to the cold-white CFL (CT of 5843 K) (See Section 3.5 for theoretical calculations). Correspondingly, at 100 lx, the MPE is 57,696 s (16 h) and 320 s, respectively. The melatonin secretion sensitivity (1.33%) at 100 lx of the studied device is 22.4 times friendlier than its counterpart cold-white CFL (29.9%) upon exposure for 1.5 h at night. 

In contrast to cold-white LEDs, the studied device exhibits 152 times retina pleasant and 15 times amicable to MSS. While, for cold white organic LEDs, the candlelight organic LED is 98 times human eye-friendly and 9.6 times friendlier to melatonin secretion. 

Moreover, the studied device is 57.6/54.9, and 6/5.2 times enhanced than warm-white LED (CT of 2704 K) and warm-white organic LED (CT of 3080 K) in terms of MPE/MSS, respectively. 

Furthermore, the fabricated candlelight organic LED is far better than incandescent bulb (CT of 2444 K) and candle light (CT of 1884 K) in prospects of both the retina damage and melatonin suppression, i.e., 52.4 times human eye-friendly and 8.6 times melatonin generation-friendly than the incandescent bulb, while 21 times human eye-friendly and 200% more melatonin generation-friendly than candle-light due to the absence of blue-emission. 

Therefore, the studied candlelight organic LED is free from flickering, scorching, glare, and, most importantly, PM 2.5, perhaps, significantly energy-efficient than any commercial luminaires.

**Figure 7:**
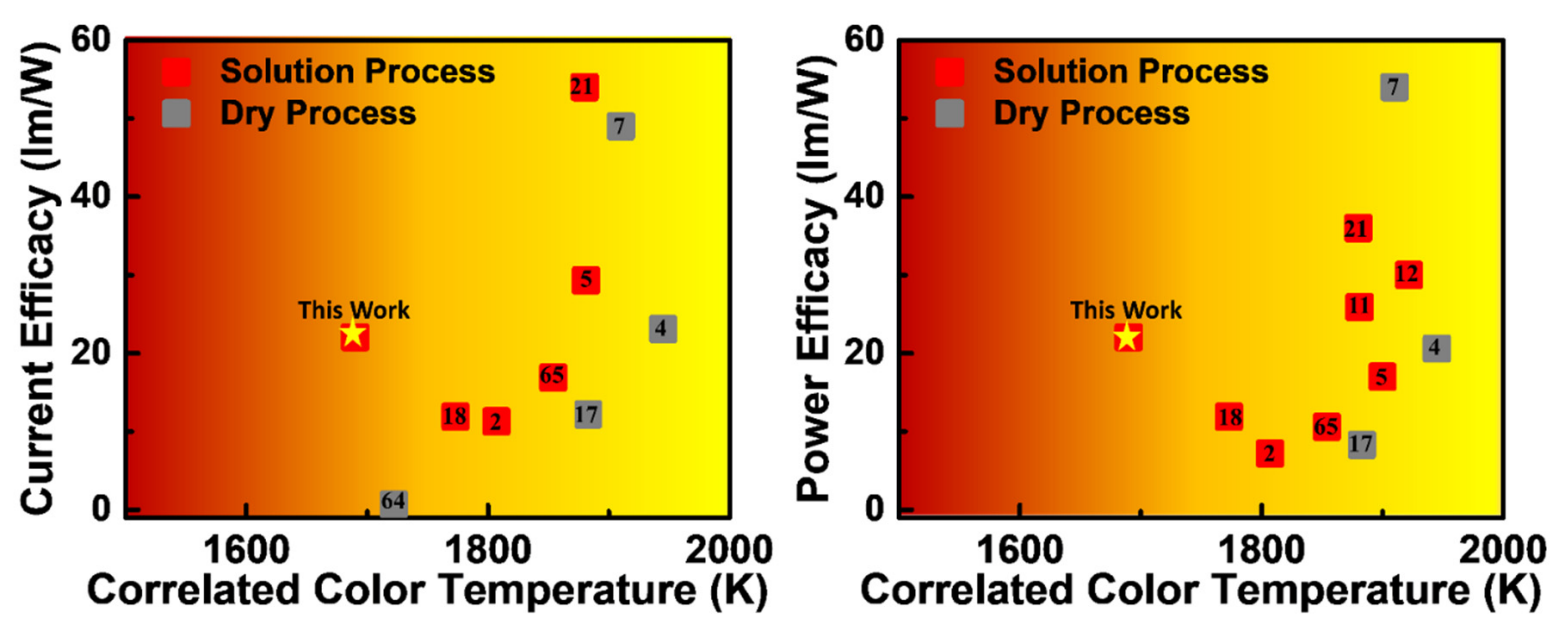
The reported color temperature (at 100 cd/m^2^) for the solution and dry-processed candlelight organic LED devices against current efficacy and power efficacy [2,4,5,7,11,12,17,18,21,64,65].

Figure 7 shows the reported color temperature (at 100 cd/m^2^) for a solution and dry-processed candlelight organic LED devices: the CT vs. CE plot displaying the lowest color temperature achieved with high CE compared to most other reports and the CT vs. PE plot displaying a high PE of 22.0 lm/W at 1690 K CT. Most devices are reported using tandem or complex device structures with more than two dopant and/or extra transporting layers. A few published papers showed candlelight organic LED fabricated via a dry process. Furthermore, a comparatively studied and reported candlelight organic LEDs showing their fabrication method, color temperature, power efficacy, current efficacy, and the respective references are revealed in Table S2. 

Therefore, this work may direct the field specialists to synthesize novel potential host materials to fabricate low-cost and energy-efficient blue-emission-free organic LED devices for solid-state lighting applications.

## 3. Materials and Methods

### 3.1. Synthesis

The as-synthesized material 3,3-bis(phenoxazin-10-ylmethyl)oxetane (BPMO) was used as the host material. The material was synthesized using silica gel column chromatography and the yield is found to be 0.24 g (42%) of yellowish crystals. The melting point is found to be at 199 °C through DSC calculation. The complete synthesis of material is described in our previously reported journal [37]. 

### 3.2. Characterization and Measurements

Thermogravimetric analysis (TGA) was conducted on TGAQ50 equipment (Verder Scientific, Haan, Germany). The TGA and DSC curves were recorded at a 10 °C/min heating rate in a nitrogen environment. A Bruker Reflex II thermos-system was used to perform differential scanning calorimetry (DSC) measurements [37]. Phosphorescence characteristic of BPMO was recorded in THF solution on a Hitachi F-7000 fluorescence spectrophotometer (Edinburgh Intruments Ltd, Livingston, United Kingdom) with a delay time of 6.25 ms at low-temperature 77 K to determine the triplet energy (E_t_). The photophysical measurement (UV-vis and photoluminescence (PL)) of the host materials BPMO and CBP was performed on Metertech SP-8001 (SHISHIN TECHNOLOGY CO., LTD., Taipei, Taiwan) and JASCO FP-6500 (JASCO FP-6500, Tokyo, Japan) . The tetrahydrofuran (THF) was used as a solvent to analyze the photophysical behavior at room temperature in quartz cuvettes. The solvent was purchased from commercial resources. The host materials BPMO and CBP solutions with solvent THF were prepared 1 mg/mL to measure UV-vis and PL. The instrument’s excitation wavelength and scan speeds were 350 nm and 10 nm/min, respectively. The electrochemical measurements (cyclic voltammetry, CV) were executed on CH instruments CH1604A electrochemical workstation (Artisan technology group, Champaign County, Illinois, United States) using three-electrode assembly, including a glassy carbon working electrode, an auxiliary platinum electrode, and a non-aqueous Ag/AgCl reference electrode. The measurement was performed at an ambient temperature under a nitrogen atmosphere in dichloromethane (DCM) using 0.1 M tetrabutylammonium perchlorate (Bu_4_NClO_4_) as the corresponding electrolyte CH-instruments CH1604A potentiostat.

### 3.3. Materials

In this research, the sputtered indium tin oxide (ITO) of glass substrates with a sheet resistance of 25 sq^−1^ was purchased from Shine Materials Technology Co. Ltd., Taiwan. The hole-transport/-injection (HTL/HIL) material, i.e., poly(3,4-ethylene-dioxythiophene)-poly-(styrenesulfonate) (PEDOT:PSS), was acquired from UniRegion Bio-tech (UR-AI 4083, Hsinchu, Taiwan). The host material 3,3-bis(phenoxazin-10-ylmethyl)oxetane (BPMO) is synthesized in our laboratory. Phenoxazine (1), 3,3-bis(chloromethyl)oxetane, THF, and potassium tert-butoxide were purchased from Aldrich and used as received. Other organic small molecules used for this work such as the one we used as a host (control part) material 4,4′-Bis(N-carbazolyl)-1,1′-biphenyl (CBP), guest materials iridium(III)bis(4-phenylthieno[3,2-c]pyridinato-N,C2′)acetylacetonate (PO-01), Tris(2-phenylquinoline)iridium(III) (Ir(2-phq)_3_, electron-transport material (ETM) 1,3,5-tris(N-phenylbenzimidazol-2-yl)benzene (TPBi), and an electron injection material lithium fluoride (LiF) were purchased from Shine Materials Technology Co. Ltd, Taiwan. Furthermore, aluminum ingots (Al) used as cathode were acquired from Showa Chemicals, Japan.

### 3.4. Device Fabrication and Characterization

The displayed highly efficient candlelight organic LEDs with very low-color temperature were fabricated in the following conventional structure: ITO (125 nm)/PEDOT:PSS (35 nm)/CBP or BPMO: 10 wt% PO-01 and x wt% Ir(2-phq)_3_ (20 nm)/TPBi (40 nm)/LiF (1 nm)/Al (200 nm). Indium tin oxide (ITO) of work function 5.2 eV sputtered on the glass substrate is used as an anode for the device. A hole-injection/-transporting material PEDOT:PSS with HOMO, LUMO 5.0, 3.3 eV, respectively, is spin-coated at 4000 rpm for the 20 s and heated for 10 min at 120 °C. Meanwhile, an emissive layer solution is prepared by dissolving the organic materials CBP, BPMO, PO-01, Ir(2-phq)_3_ in tetrahydrofuran (THF) and sonicated for 30 min at 60 °C. Once the solutions are completely dissolved and cooled, they are filtered separately. Two distinct EML solutions are prepared, one with CBP as a host and the other as BPMO. 10 wt% PO-01 and different concentrations of Ir(2-phq)_3_ such as 7.5, 10, 12.5, and 15 wt% were mixed in two host solutions and named as EML1 (with CBP) and EML2 (BPMO), keeping CBP as a control part for the experiment. The as-prepared EMLs are spin-coated at 2500 rpm at ambient temperature for 20 s on the pre-deposited PEDOT:PSS, and the devices are kept in sample boxes for further processes. The entire spin-coating process is performed in an inert environment of the glove box. Subsequently, the devices are transferred to a pre-loaded thermal evaporation chamber. Once the vacuum is reached below 10^–6^ torr, TPBi, LiF, and Al deposition is performed for the defined layer thicknesses. Further, the fabricated devices are kept in a mini-chamber of the glove box and taken for testing one at a time. The current-voltage-luminance (J-V-L) measurement is done by a Keithley source measurement unit (Keithley 2400). The CIE chromatic coordinates, electroluminescence spectra, and luminance are determined using a Photo Research PR-655 spectrum scan and CS100A luminance meter. The device emission area is defined as the overlapping area of the visible cathode, and the anode is used as 9 mm^2^. All the measured luminance is taken in forward directions. The entire testing process is performed in a closed dark room in an ambient environment.

### 3.5. Theoretical Calculation of MSS and MPE

#### 3.5.1. Maximum Permissible Exposure-Limit (MPE)

The maximum permissible exposure-limit (MPE) presented by the international Commission on Non-radiation Protection Council (ICNIRP) [66] is used to quantify the blue light hazards, which can be calculated as following:

(1) $$MPE=\frac{100}{E_{B}}$$

where *E*_B_ is the photo-retinitis or blue light hazard weighted radiation (W/m^2^) [12,67,68].

#### 3.5.2. Melatonin Suppression Sensitivity

The melatonin suppression sensitivity (MSS) was presented by Prof. Jou [69,70], which can be calculated by the following formula:

(2) $$MSS=\frac{S_{\mathrm{LC}}\left(\lambda \right)}{S_{\mathrm{LC}}\left(480\right)}\times 100\%$$

where *S*_LC_ is the melatonin suppression spectrum per lux for a given polychromatic light, relative to that for a reference blue light of 480 nm.

## 4. Conclusions

We reported a solution-processable candlelight organic LED with a very low color-temperature via a simpler device architecture. The device consists of a phenoxazine-based host BPMO along with an orange-red and a yellow dye, Ir(2-phq)_3_ and PO-01, respectively. The study shows a color temperature of 1690 K, which is significantly lower than the oil lamps (1800 K), candles (1900 K), and incandescent bulbs (2444 K). Furthermore, at 100 lx, a record-breaking maximum permissible exposure limit of 57,696 s is obtained along with 1.33% melatonin suppression sensitivity upon exposure for 1.5 h at night. Moreover, BPMO-based candlelight organic LED device enhanced a 200, 120, and 120% in PE, CE, and EQE at 100 cd/m^2^, respectively, concerning CBP. The fundamental elements underlying better device efficiencies have excellent electron-blocking abilities, suitable HOMO, LUMO, and triple energy levels, decreased hole-injection barrier between host and HIL, and substantially confined light-emitting excitation to the required recombination zone; moreover, the BPMO-based candlelight organic LED. This work will enable the fabrication of highly efficient candlelight organic LED lighting devices with the feasibility of solution processes.

## Figures and Tables

**Table 1:** A comprehensive list of photophysical and electrochemical properties of hosts BPMO and CBP for emission wavelength, melting temperature, glass-transition temperature, decomposition temperature, HOMO, LUMO, singlet/triplet energies, and bandgap.

Material	λ_em_ ^a^ (nm)	*T*m ^b^ (°C)	*T*_g_ ^c^ (°C)	*T*_d_ ^d^ (°C)	HOMO ^e^ (eV)	LUMO ^e^ (eV)	HOMO ^f^ (eV)	LUMO ^f^ (eV)	E_t_^g^ (eV)	E_s_^g^ (eV)	E_t_^h^ (eV)	E_s_^h^ (eV)	Band Gap ^i^ (eV)	Refs.
**BPMO**	393	199	63	340	5.12	0.71	5.39	1.54	3.02	3.70	2.87	3.44	3.85	[37]
**CBP**	369		62	320	-	-	6.0	2.9	2.56	3.49	2.6	-	3.1	[38,39,40]

^a^ Photoluminescence peak; ^b^ melting temperature; ^c^ glass transition temperature; ^d^ decomposition temperature; ^e^ HOMO and LUMO were calculated by DFT. ^f^ the redox potential obtained using a cyclic voltammetry (CV) technique gives HOMO and LUMO. ^g^ Calculated triplet (E_t_) and singlet (E_s_) energy by DFT. ^h^ Calculated triplet (E_t_) and singlet (E_s_) energy by UV-vis and low-temperature PL. ^i^ Optical energy band gap.

**Table 2:** Power efficacy (PE), current efficacy (CE), EQE, color-temperature (CT) of studied BPMO- and CBP-based candlelight organic LED using (at 10 wt%) yellow (PO-01) and orange-red (Ir(2-phq)_3_) emitters.

Hosts.	Emitter (wt%)		DV (V)	OV (V)	PE (lm/W)	CE (cd/A)	EQE (%)	CT (K)	CIE	L_max_ (cd/m^2^)
	PO-01	Ir(2-phq)_3_		@100/1000/10,000/max. (cd/m^2^)						
**BPMO**	10	10	2.8	3.2/4.1/6.4/2.9	22.0/16.9/5.6/23.7	22.4/21.6/11.6/ 22.4	10.2/9.6/5.3/ 10.2	1690/1707/1786	(0.58, 0.42)/(0.58, 0.42)/(0.57, 0.43)	14,950
**CBP**	10	10	3.2	4.1/5.7/-/3.5	7.4/3.9/-/9.6	10.3/7.4/-/11.7	4.7/3.2/-/6.8	1768/1782/-	(0.57, 0.42)/(0.57, 0.42)/-	8393

**Table 3:** Effects of ETL (TPBi) thickness on power efficacy (PE), current efficacy (CE), EQE, color-temperature (CT) of studied BPMO- and CBP-based candlelight organic LED using (at 10 wt%) yellow (PO-01) and orange-red (Ir(2-phq)_3_) emitters.

ETL (nm)	DV (V)	OV (V)	PE (lm/W)	CE (cd/A)	EQE (%)	CT (K)	CIE	L_max_ (cd/m^2^)
		@100/1000/10,000/max. (cd/m^2^)						
45	2.8	3.2/4.1/6.4/ 2.9	22.0/16.9/5.6/23.1	22.4/21.6/11.6/22.1	10.2/9.6/5.3/9.5	1690/1707/1786/-	(0.58, 0.42)/(0.58, 0.42)/(0.57, 0.43)	14,950
50	2.8	3.2/4.1/6.6	24.7/17.9/5.4/24.8	24.8/23.3/11.3/28.8	10.6/9.9/-/10.6	1785/1795/1898/-	(0.57, 0.43)/(0.57, 0.43)/-	13,520
55	2.9	3.3/4.6/7.1	22.9/10.4/4.9/23.7	24.2/15.3/11/24.2	10.7/6.7/-/10.7	1732/1744/1837/-	(0.57, 0.43)/(0.57, 0.43)/-	13,250

**Table 4:** Comparison between the spectrum (350–780 nm), color temperature (CT), melatonin suppression sensitivity (MSS) (1.5 h exposure), and maximum permissible exposure limit (MPE) of the studied very low color temperature candlelight organic LED and the commercial luminaires.

Light Source	Spectrum	CT (K)	MSS (%)	MPE (s) @100 lx
This work	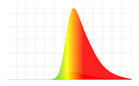	1690	1.33	57,696
Candle Light	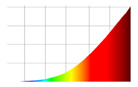	1884	4.0	2750
Incandescent Bulb	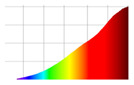	2444	11.5	1100
Warm-white LED	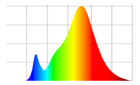	2704	8.0	1000
Warm-white organic LED	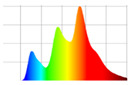	3080	6.9	1050
Cold-white organic LED	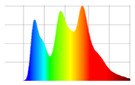	4034	12.8	590
Cold-white LED	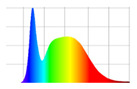	5549	19.8	380
Cold-white CFL	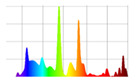	5843	29.9	320

## Supplementary Materials

The following are available online, Figure S1: cyclic voltammetry (CV) curve showing one-step oxidation of the host BPMO, Figure S2: the chemical structure of HIL (PEDOT:PSS), host (BPMO and CBP), yellow emitter (PO-01), orange-red emitter (Ir(2-phq)_3_), and ETL (TPBi) materials incorporated in this research, Figure S3: the studied BPMO-based candlelight organic LEDs with (at 10 wt%) yellow and (at 7.5, 10 and 12.5 wt%) orange-red emitter, Figure S4: the studied CBP-based candlelight organic LEDs with (at 10 wt%) yellow and (at 7.5, 10 and 12.5 wt%) orange-red emitter, Table S1: power efficacy (PE), current efficacy (CE), EQE, color-temperature (CT) of studied BPMO- and CBP-based candlelight organic LED, Table S2: a comparative study of the studied and reported candlelight organic LEDs showing the fabrication method, color temperature, power efficacy, current efficacy.

## Sample Availability

Samples of the compounds BPMO are available from the authors.
