# Supplementary material for: Highly Efficient Candlelight Organic Light-Emitting Diode with a Very Low Color Temperature
Source: Molecules. 2021 Dec 13;26(24):7558. doi: 10.3390/molecules26247558 (PMC8707034; doi:10.3390/molecules26247558)
Supplement: Supplementary file 1 [file molecules-26-07558-s001.zip › molecules-1487974-supplementary.pdf]

# Electronic Supporting Information

# Highly Efficient Candlelight Organic Light-Emitting Diode with a Very Low Color Temperature

Shahnawaz[1], Iram Siddiqui[1], Mangey Ram Nagar[1], A. Choudhury[1], Jin-Ting Lin[1], Dovydas Blazevicius[2], Gintare Krucaite[2], Saulius Grigalevicius[2*], Jwo-Huei Jou[1*]

[1]Department of Materials Science and Engineering, National Tsing Hua University, Hsinchu, 30044, Taiwan ROC

[2]Department of Polymer Chemistry and Technology, Kaunas University of Technology, Lithuania.

*Corresponding Author: Jwo-Huei Jou, Email: jjou@mx.nthu.edu.tw and Saulius Grigalevicius, Email: saulius.grigalevicius@ktu.lt

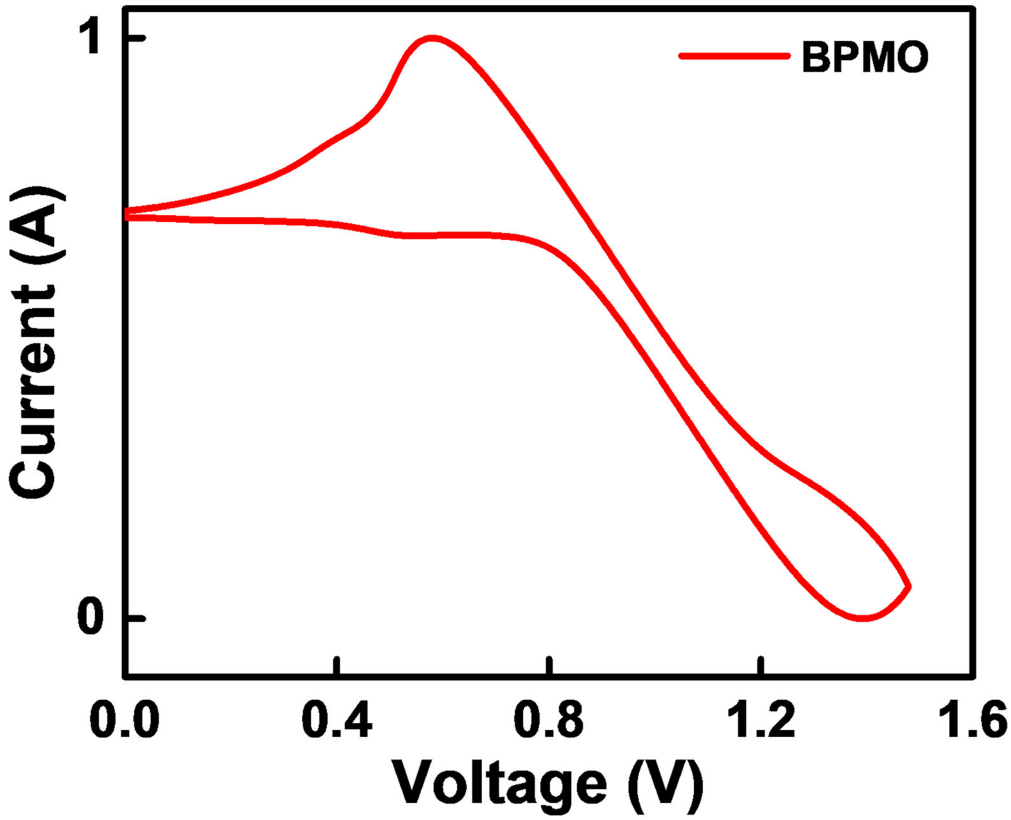


**Figure S1.** Cyclic voltammetry (CV) curve showing one-step oxidation of the host BPMO. The host material and electrolyte tetrabutylammonium perchlorate were dissolved in the solvent dichloromethane (DCM). The scan rate was set to 100 mV/s. The HOMO value was calculated

using $E_{HOMO} = -(E_{1/2}^{ox} + 4.8)$. The energy of LUMO was estimated by subtracting the bandgap from the HOMO energy level using ($E_{LUMO} = -E_{HOMO} + E_g$).

## Scheme

## S1. Synthesis

The as-synthesized material 3,3-bis(phenoxazin-10-ylmethyl)oxetane (**BPMO**) was used as the host material. The material was synthesized using silica gel column chromatography and the yield is found to be 0.24 g (42%) of yellowish crystals. The melting point is found to be at 199 ℃ through DSC calculation. The complete synthesis of material is described in our previously reported journal[1].

## S2. Characterization and measurements

Thermogravimetric analysis (TGA) was conducted on TGAQ50 equipment. The TGA and DSC curves were recorded at a 10 ºC/min heating rate in a nitrogen environment. A Bruker Reflex II thermos-system was used to perform differential scanning calorimetry (DSC) measurements[1]. Phosphorescence characteristic of BPMO was recorded in THF solution on a Hitachi F-7000 fluorescence spectrophotometer with a delay time of 6.25 ms at low-temperature 77 K to determine the triplet energy ($E_t$). The photophysical measurement (UV-vis and photoluminescence (PL)) of the host materials BPMO and CBP was performed on Shimadzu UV-2450 and Perkin Elmer LS55. The tetrahydrofuran (THF) was used as a solvent to analyze the photophysical behavior at room temperature in quartz cuvettes. The solvent was purchased from commercial resources. The host materials BPMO and CBP solutions with solvent THF were prepared 1 mg/ml to measure UV-vis and PL. The instrument's excitation wavelength and scan speeds were 350 nm and 10 nm/minute, respectively. The electrochemical measurements (cyclic voltammetry (CV)) were executed in an electrochemical workplace using three-electrode assembly, including a glassy carbon working electrode, an auxiliary platinum electrode, and a non-aqueous Ag/AgCl reference electrode. The measurement was performed at an ambient temperature under a nitrogen atmosphere in dichloromethane (DCM) using 0.1 M tetrabutylammonium perchlorate (Bu4NClO4) as the corresponding electrolyte CH-instruments CH1604A potentiostat.

## S3. Materials

In this research, the sputtered indium tin oxide (ITO) of glass substrates with a sheet resistance of 25 sq$^{-1}$ was purchased from Shine Materials Technology Co. Ltd., Taiwan. The hole-transport/-injection (HTL/HIL) material, i.e., poly(3,4-ethylene-dioxythiophene)-poly-(styrenesulfonate) (PEDOT:PSS), was acquired from UniRegion Bio-tech (UR-AI 4083), Taiwan. The host material 3,3-bis(phenoxazin-10-ylmethyl)oxetane (BPMO) is synthesized in our laboratory. Phenoxazine (1), 3,3-bis(chloromethyl)oxetane, THF, and potassium tert-butoxide were purchased from Aldrich and used as received. Other organic small molecules used for this work such as the one we used as a host (control part) material 4,4′-Bis(N-carbazolyl)-1,1′-biphenyl (CBP), guest materials iridium(III)bis(4-phenylthieno[3,2-c]pyridinato-N,C2′)acetylacetonate (PO-01), Tris(2-phenylquinoline)iridium(III) (Ir(2-phq)$_3$, electron-transport material (ETM) 1,3,5-tris(N-phenylbenzimidazol-2-yl)benzene (TPBi), and an electron injection material lithium fluoride (LiF) were purchased from Shine Materials Technology Co. Ltd, Taiwan. Furthermore, aluminum ingots (Al) used as cathode were acquired from Showa Chemicals, Japan.

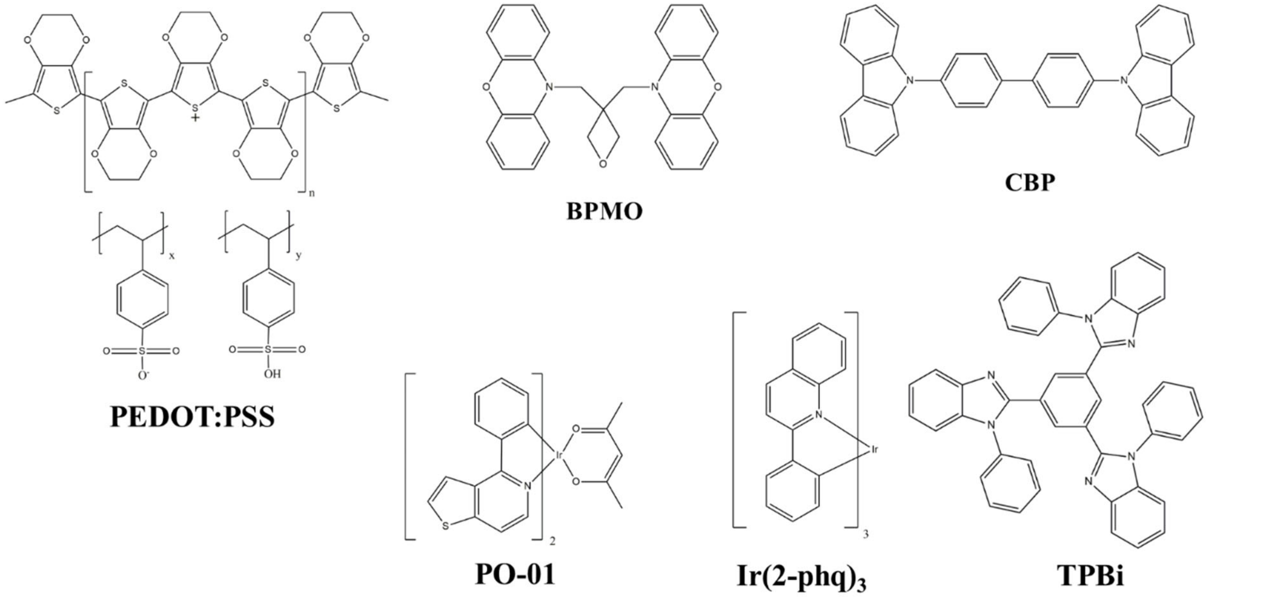


**Figure S2.** The chemical structure of HIL (PEDOT:PSS), host (BPMO and CBP), yellow emitter (PO-01), orange-red emitter (Ir(2-phq)$_3$), and ETL (TPBi) materials incorporated in this research.

## S4. Device fabrication and characterization

The displayed highly efficient candlelight organic LEDs with very low-color temperature were fabricated in the following conventional structure: ITO (125 nm)/PEDOT:PSS (35 nm)/CBP or

BPMO: 10 wt% PO-01 and x wt% Ir(2-phq)$_3$ (20 nm)/TPBi (40 nm)/LiF (1nm)/Al (200 nm). Indium tin oxide (ITO) of work function 5.2 eV sputtered on the glass substrate is used as an anode for the device. A hole-injection/-transporting material PEDOT:PSS with HOMO, LUMO 5.0, 3.3 eV, respectively, is spin-coated at 4000 rpm for the 20 s and heated for 10 minutes at 120 ºC. Meanwhile, an emissive layer solution is prepared by dissolving the organic materials CBP, BPMO, PO-01, Ir(2-phq)$_3$ in tetrahydrofuran (THF) and sonicated for 30 minutes at 60 ºC. Once the solutions are completely dissolved and cooled, they are filtered separately. Two distinct EML solutions are prepared, one with CBP as a host and the other as BPMO. 10 wt% PO-01 and different concentrations of Ir(2-phq)$_3$ such as 7.5, 10, 12.5, and 15 wt% were mixed in two host solutions and named as EML1 (with CBP) and EML2 (BPMO), keeping CBP as a control part for the experiment. The as-prepared EMLs are spin-coated at 2500 rpm at ambient temperature for 20 s on the pre-deposited PEDOT:PSS, and the devices are kept in sample boxes for further processes. The entire spin-coating process is performed in an inert environment of the glove box. Subsequently, the devices are transferred to a pre-loaded thermal evaporation chamber. Once the vacuum is reached below $10^{-6}$ torr, TPBi, LiF, and Al deposition is performed for the defined layer thicknesses. Further, the fabricated devices are kept in a mini-chamber of the glove box and taken for testing one at a time. The current-voltage-luminance (J-V-L) measurement is done by a Keithley source measurement unit (Keithley 2400). The CIE chromatic coordinates, electroluminescence spectra, and luminance are determined using a Photo Research PR-655 spectrum scan and CS100A luminance meter. The device emission area is defined as the overlapping area of the visible cathode, and the anode is used as 9 mm$^2$. All the measured luminance is taken in forward directions. The entire testing process is performed in a closed dark room in an ambient environment.

## S5. Theoretical calculation of MSS and MPE

### (a) Maximum permissible exposure-limit (MPE)

The maximum permissible exposure-limit (MPE) presented by the international Commission on Non-radiation Protection Council (ICNIRP)[2] is used to quantify the blue light hazards, which can be calculated as following:

$$\boldsymbol{MPE = \frac{100}{E_B}}$$

where $E_B$ is the photo-retinitis or blue light hazard weighted radiation ($W/m^2$)[3–5].

**(b) Melatonin suppression sensitivity**

The melatonin suppression sensitivity (MSS) was presented by Prof. Jou [6,7], which can be calculated by the following formula:

$$\boldsymbol{MSS} = \frac{\boldsymbol{S_{LC}(\lambda)}}{\boldsymbol{S_{LC}(480)}} \times \mathbf{100\%}$$

where $S_{LC}$ is the melatonin suppression spectrum per lux for a given polychromatic light, relative to that for a reference blue light of 480 nm.

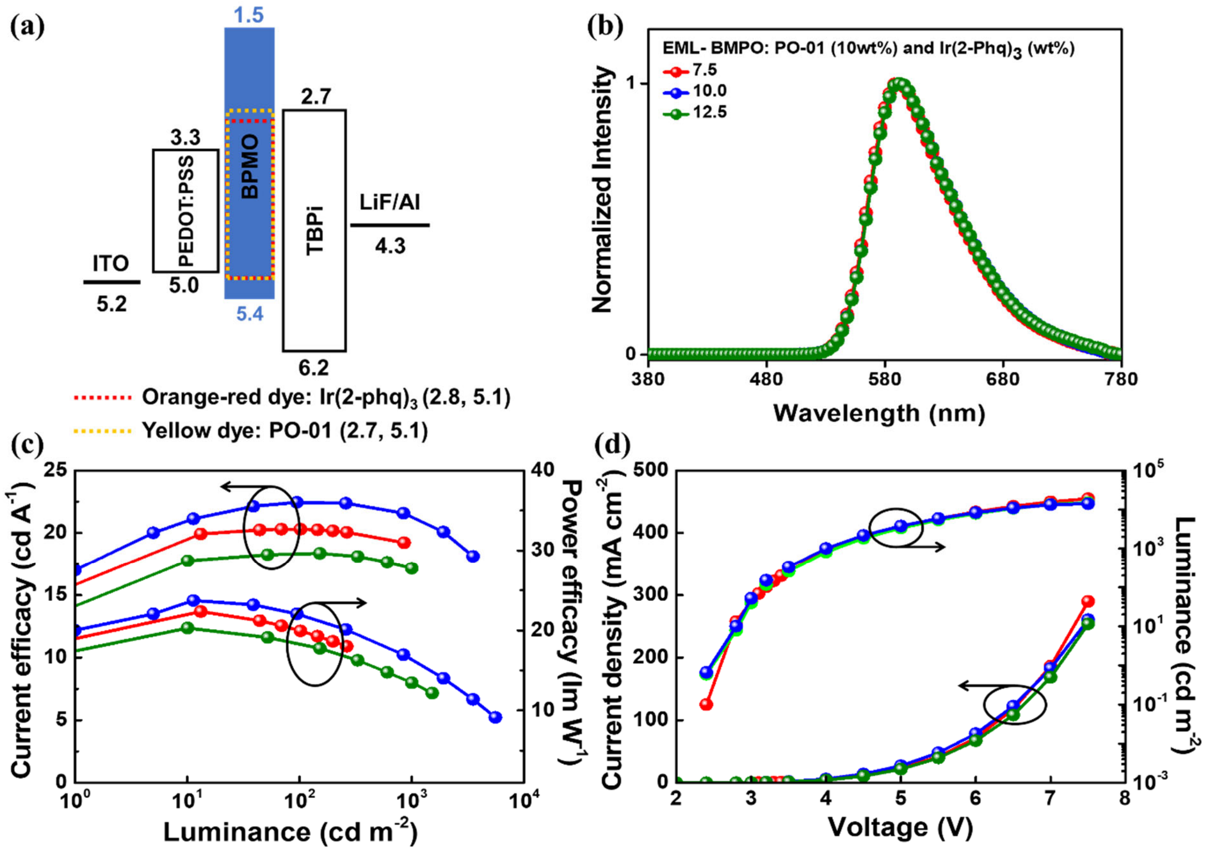


**Figure S3.** The studied BPMO-based candlelight organic LEDs with (at 10 wt%) yellow and (at 7.5, 10 and 12.5 wt%) orange-red emitter showing (a) the electroluminescent spectra, (b) variation of EQE with respect to luminance, (c) luminance-voltage-current density (L–V–J), (d) power efficacy-luminance-current-efficacy curve. The 10 wt% orange-red emitter concentration shows best efficiencies.

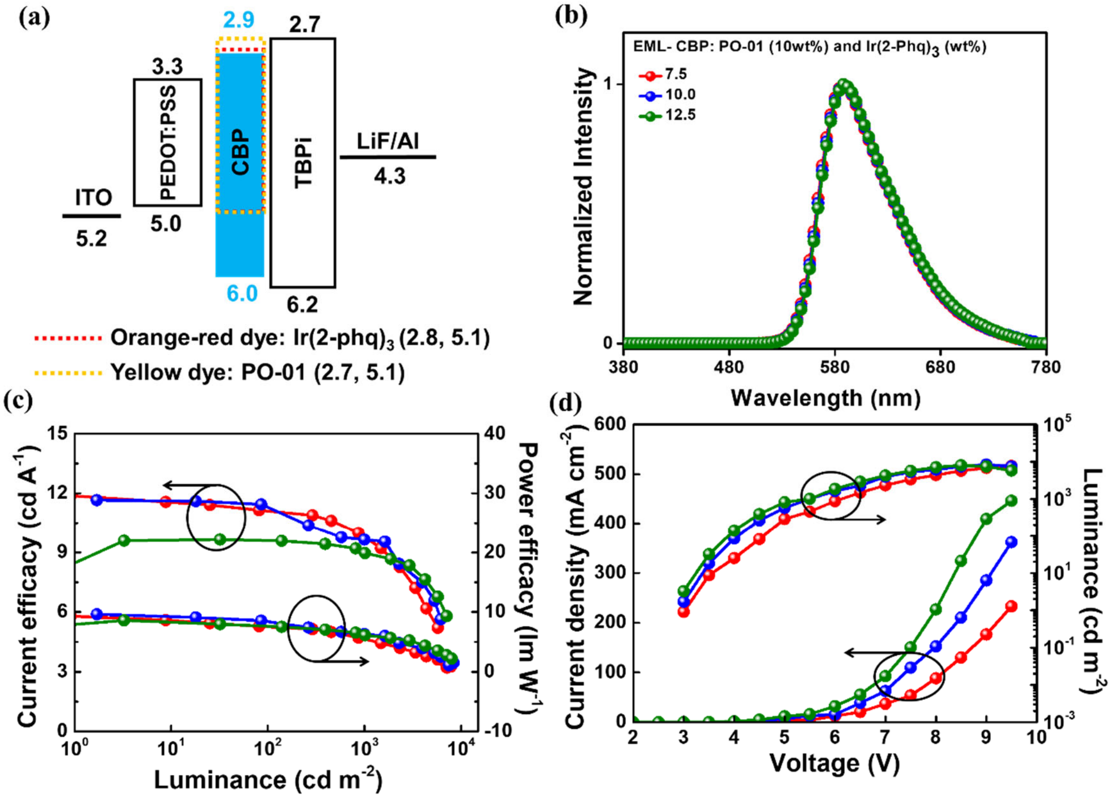


**Figure S4.** The studied CBP-based candlelight organic LEDs with (at 10 wt%) yellow and (at 7.5, 10 and 12.5 wt%) orange-red emitter showing (a) the electroluminescent spectra, (b) variation of EQE with respect to luminance, (c) luminance-voltage-current density (L–V–J), (d) power efficacy-luminance-current-efficacy curve. The 10 wt% orange-red emitter concentration shows best efficiencies.

**Table S1.** Power efficacy (PE), current efficacy (CE), EQE, color-temperature (CT) of studied BPMO- and CBP-based candlelight organic LED using (at 10 wt%) yellow (PO-01) and (at 7.5, 10, 12.5, 15 wt%) orange-red (Ir(2-phq)3) emitters

| Hosts | Dopants (wt%) | | DV (V) | OV (V) | PE (lm/W) | CE (cd/A) | EQE (%) | CT (K) | CIE | $L_{max}$ (cd/m$^2$) |
|---|---|---|---|---|---|---|---|---|---|---|
| | PO-01 | Ir(2-phq)3 | | @100/1,000/10,000/max. (cd/m$^2$) | | | | | | |
| BPMO | 10 | 7.5 | 2.8 | 3.2/ 4.1 /6.2/2.9 | 20/ 14.8/ 5.8/ 22.1 | 20.3/ 19/ 11.5/ 20.3 | 9.0/ 8.4/ 5/ 9.2 | 1,30/1742 /1826 | (0.57, 0.43)/ (0.57, 0.43)/ (0.57, 0.43) | 19,130 |
| | | 10 | 2.8 | 3.2/ 4.1 /6.4/2.9 | 22.04/ 16.9/ 5.6/ 23.7 | 22.4/ 21.6/ 11.6/ 22.4 | 10.2/ 9.6/ 5.3/ 10.2 | 1690/1707 / 1786 | (0.58, 0.42) /(0.58, 0.42) / (0.57, 0.43) | 14,950 |

| | | | | | | | | | |
|---|---|---|---|---|---|---|---|---|---|
| | | 12.5 | 3 | 3.1/ 4/ 6.3/ 2.8 | 18.5/ 13.5/ 4.8/ 19.2 | 18.3/ 17.1/ 9.6/ 18.3 | 8.2/ 7.7/ 4.1/ 8.2 | 1696/1706 / 1819 | (0.58, 0.42)/ (0.58, 0.42)/ (0.57, 0.43) | 14,160 |
| | | 15 | 2.8 | 3.1/ 4/ 6.2/ 2.8 | 18.3/ 13.4/ 4.9/ 19.9 | 18.2/ 17.2/ 9.7/ 18.2 | 8.2/ 7.7/ 4.3/ 8.5 | 1705/1715 / 1796 | (0.58, 0.42)/ (0.58, 0.42)/ (0.57, 0.43) | 15,350 |
| | | | | | | | | | | |
| **CBP** | 10 | 7.5 | 3.2 | 4.6/ 6.6 /-/ 4.3 | 8.1/ 4.8/ - / 9.3 | 11.4/ 9.2/- / 11.8 | 4.7/ 3.9/-/ 5.0 | 1795/1804 /- | (0.56, 0.43)/ (0.56, 0.43)/- | 7,979 |
| | | 10 | 3.2 | 4.1/ 5.7 /-/ 3.5 | 7.4/ 3.9/ - / 9.6 | 10.3/ 7.4/- / 11.7 | 4.7/ 3.2/-/ 6.8 | 1768/1782 /- | (0.57, 0.42)/ (0.57, 0.42)/- | 8,393 |
| | | 12.5 | 3.2 | 4/ 5.4/ -/ 3.5 | 7.5/ 4.3/ - / 8.6 | 9.5/ 7.6/-/ 9.5 | 4.1/ 3.3/-/ 4.1 | 1742/1757 /- | (0.57, 0.42)/ (0.57, 0.42)/- | 8,037 |
| | | 15 | 3.1 | 3.8/ 5.3 /-/ 3.3 | 7.6/ 4.1/ - / 7.6 | 9.3/ 7.3/-/ 9.3 | 4.0/ 3.7/-/ 4.0 | 1723/1738 /- | (0.57, 0.42)/ (0.57, 0.42)/- | 7,341 |

.

## S6. The previous studied and reported candlelight organic LEDs

A comparatively reported candlelight organic LEDs showing their fabrication method, color temperature, power efficacy, current efficacy and the respective references are revealed in Table S2. Most devices are reported using tandem or complex device structures with more than two dopant and/or extra transporting layers. A few published papers show candlelight organic LED fabricated via a dry process.

In 2012, Jou et al. reported a physiologically friendly low color-temperature night light by doping a deep red and yellow dopant into a blue light-emitting host. The device shows PE of 11.9 lm/W and CE of 11.9 cd/A with a color-temperature of 1773 K [15]. In 2019, Korshunov et al. reported a dry-processed candle light-style organic LED with a low color temperature (1,722 K) showing a CE of 0.7 cd/A [8]. Singh et al., in 2018, demonstrated a wet-processed single emissive layer-based low color-temperature organic LED showing a 10.6 lm/W PE and 16.9 cd/A CE with color-temperature 1854 K [9]. In 2014, Gong et al. reported an aggregation-induced emission, thermally stable and mechanochromic emitters for low-temperature organic LED. The device shows PE of 8.3 lm/W and CE of 12.2 cd/A with a 1883 K color-temperature [13]. In the same year, Hu et al. demonstrated a hybrid low color temperature organic LED via dry process showing a color-temperature of 1910 K, a highest 54 lm/W PE, and 49 cd/A CE. The device was fabricated using two different co-hosts approaches, and EMLs composed of three primary color emitters [12]. Jou et al., in 2011, reported a low color-temperature organic LED using a blend interlayer between two

emissive layers (blue and red emitters). The device shows a 36 lm/W PE and 54 cd/A CE with a 1880 K color-temperature [16].

**Table S2.** A comparative study of the studied and reported candlelight organic LEDs showing the fabrication method, color temperature, power efficacy, current efficacy, and references.

| Fabrication Method | Color Temperature (K) | Power Efficacy (lm/W) | Current Efficacy (cd/A) | Ref. |
|---|---|---|---|---|
| Wet | 1,690 | 22.0 | 22.4 | **This Work** |
| Dry-Process | 1,722 | - | 0.7 | [8] |
| Wet | 1,854 | 10.6 | 16.9 | [9] |
| Dry-process | 1,945 | 20.6 | 23.1 | [10] |
| Wet | 1,922 | 30 | - | [5] |
| Wet | 1,807 | 7.2 | 11.3 | [11] |
| Dry-process | 1,910 | 54 | 49 | [12] |
| Dry-process | 1,883 | 8.3 | 12.2 | [13] |
| Wet | 1900 | 17 | - | [14] |
| Wet | 1,773 | 11.9 | 11.9 | [15] |
| Wet | 1880 | 36 | 54 | [16] |
| Wet | 1881 | 25.9 | 29.3 | [17] |

## References


1. Blazevicius, D.; Krucaite, G.; Shahnawaz, S.; Swayamprabha, S.S.; Zaleckas, E.; Jou, J.-H.; Grigalevicius, S. Easily synthesized and cheap carbazole- or phenoxazine-based hosts for efficient yellow phosphorescent OLEDs. *Opt. Mater. (Amst).* **2021**, *118*, 111251,

doi:10.1016/j.optmat.2021.111251.
2. Protection, R. and I.-I. Protection, Guidelines on Limits of Exposure for Broad-band Incoherent Optical Radiation (0.38 to 3 μm). Health Phys, **1997**. 73(3): p. 539-597. - Google Search Available online: https://www.google.com/search?q=Protection%2C+R.+and+I.-I.+Protection%2C+Guidelines+on+Limits+of+Exposure+for+Broad-band+Incoherent+Optical+Radiation+(0.38+to+3+μm).+Health+Phys%2C+1997.+73(3)%3A+p.+539-597.&rlz=1C1CHBF_enTW878TW879&oq=Protection%2C+R.+and+I.-I.+Protection%2C+Guidelines+on+Limits+of+Exposure+for+Broad-band+Incoherent+Optical+Radiation+(0.38+to+3+μm).+Health+Phys%2C+1997.+73(3)%3A+p.+539-597.&aqs=chrome..69i57.2101j0j7&sourceid=chrome&ie=UTF-8 (accessed on Jul 28, 2021).
3. Bullough, J.D. The Blue-Light Hazard: A Review. *http://dx.doi.org/10.1080/00994480.2000.10748312* **2013**, *29*, 6–14, doi:10.1080/00994480.2000.10748312.
4. JB, O.; M, K.; LL, P. Low-energy light bulbs, computers, tablets and the blue light hazard. *Eye (Lond).* **2016**, *30*, 230–233, doi:10.1038/EYE.2015.261.
5. Jou, J.-H.; Singh, M.; Su, Y.-T.; Liu, S.-H.; He, Z.-K. Blue-hazard-free Candlelight OLED. *J. Vis. Exp.* **2017**, *2017*, 54644, doi:10.3791/54644.
6. US20120303282A1 - Melatonin Suppression Extent Measuring Device - Google Patents Available online: https://patents.google.com/patent/US20120303282 (accessed on Jul 28, 2021).
7. Jou, J.H.; He, Z.K.; Su, Y.T.; Tsai, Y.F.; Wu, C.H. Approach for fabricating healthy OLED light sources with visual quality and energy-saving character. *Org. Electron.* **2016**, *38*, 396–400, doi:10.1016/J.ORGEL.2016.09.005.
8. Korshunov, V.M.; Chmovzh, T.N.; Knyazeva, E.A.; Taydakov, I. V.; Mikhalchenko, L. V.; Varaksina, E.A.; Saifutyarov, R.S.; Avetissov, I.C.; Rakitin, O.A. A novel candle light-style OLED with a record low colour temperature. *Chem. Commun.* **2019**, *55*, 13354–13357, doi:10.1039/C9CC04973H.
9. Singh, M.; Jou, J.H.; Sahoo, S.; Sujith, S.; He, Z.K.; Krucaite, G.; Grigalevicius, S.; Wang, C.W. High light-quality OLEDs with a wet-processed single emissive layer. *Sci. Reports 2018 81* **2018**, *8*, 1–9, doi:10.1038/s41598-018-24125-4.
10. Sun, Q.; Hu, Y.; Dai, Y.; Ma, D. Low color-temperature, high color rendering index hybrid white organic light-emitting diodes by the effective control of exciton

recombination zone. *J. Mater. Chem. C* **2017**, *5*, 8022–8026, doi:10.1039/C7TC02829F.
11. Jou, J.H.; Singh, M.; Song, W.C.; Liu, S.H. Printable candlelight-style organic light-emitting diode. *IOP Conf. Ser. Mater. Sci. Eng.* **2017**, *215*, 012022, doi:10.1088/1757-899X/215/1/012022.
12. Hu, Y.; Zhang, T.; Chen, J.; Ma, D.; Cheng, C.H. Hybrid Organic Light-Emitting Diodes with Low Color-Temperature and High Efficiency for Physiologically-Friendly Night Illumination. *Isr. J. Chem.* **2014**, *54*, 979–985, doi:10.1002/IJCH.201400066.
13. Gong, Y.; Liu, J.; Zhang, Y.; He, G.; Lu, Y.; Fan, W. Bin; Yuan, W.Z.; Sun, J.Z.; Zhang, Y. AIE-active, highly thermally and morphologically stable, mechanochromic and efficient solid emitters for low color temperature OLEDs. *J. Mater. Chem. C* **2014**, *2*, 7552–7560, doi:10.1039/c4tc00915k.
14. Jou, J.H.; Hsieh, C.Y.; Tseng, J.R.; Peng, S.H.; Jou, Y.C.; Hong, J.H.; Shen, S.M.; Tang, M.C.; Chen, P.C.; Lin, C.H. Candle Light-Style Organic Light-Emitting Diodes. *Adv. Funct. Mater.* **2013**, *23*, 2750–2757, doi:10.1002/ADFM.201203209.
15. Jou, J.H.; Tang, M.C.; Chen, P.C.; Wang, Y.S.; Shen, S.M.; Chen, B.R.; Lin, C.H.; Wang, W. Ben; Chen, S.H.; Chen, C.T.; et al. Organic light-emitting diode-based plausibly physiologically-friendly low color-temperature night light. *Org. Electron.* **2012**, *13*, 1349–1355, doi:10.1016/j.orgel.2012.03.035.
16. Jou, J.H.; Chen, S.H.; Shen, S.M.; Jou, Y.C.; Lin, C.H.; Peng, S.H.; Hsia, S.P.; Wang, C.W.; Chen, C.C.; Wang, C.C. High efficiency low color-temperature organic light-emitting diodes with a blend interlayer. *J. Mater. Chem.* **2011**, *21*, 17850–17854, doi:10.1039/C1JM12339D.
17. Jou, J.H.; Su, Y.T.; Liu, S.H.; He, Z.K.; Sahoo, S.; Yu, H.H.; Chen, S.Z.; Wang, C.W.; Lee, J.R. Wet-process feasible candlelight OLED. *J. Mater. Chem. C* **2016**, *4*, 6070–6077, doi:10.1039/C6TC01968D.
